# Advances in Molecular Microbiology—From Recent Advances to the Future

**DOI:** 10.3390/cimb48010051

**Published:** 2025-12-31

**Authors:** Bruce S. Seal

**Affiliations:** Biology Program and Honors College, Oregon State University—Cascades, 1500 SW Chandler Avenue, Bend, OR 97702, USA; bruce.seal@osucascades.edu

The Molecular Microbiology section of *Current Issues in Molecular Biology* publishes original research and review articles on microbes, including bacteria, archaea, eukaryotic microorganisms, and viruses. Published investigations address the fundamental mechanisms of molecular microbiology, encompassing areas ranging from basic research to translational or applied research. Areas of interest include mechanisms of antimicrobial resistance, vaccine development, the evolution of host–pathogen interactions, microbial ecology, comparative microbiology of various hosts, and microbial genomics.

Recent developments in molecular microbiology include the continuation of the search for alternatives to antibiotics, utilizing bacteriophage therapy [1,2]. This is especially true for the ESKAPE pathogens, including *Enterococcus faecium*, *Staphylococcus aureus*, *Klebsiella pneumoniae*, *Acinetobacter baumannii*, *Pseudomonas aeruginosa*, and *Enterobacter* spp. This group of bacteria comprises organisms that are highly predisposed to antibiotic resistance; they are therefore difficult to treat with traditional antibiotics to achieve effective infection control [3]. Although newly developed antibiotics, such as cefiderocol, have shown promise, resistance to this siderophore has been demonstrated among certain cohort groups [4]. Consequently, research into developing personalized therapies using bacteriophages as an alternative treatment in clinical settings could be an important aspect of future treatment approaches [5]. To achieve the goal of utilizing bacteriophages as antimicrobials, it will be necessary to continue supporting the isolation and characterization of bacteriophages from a variety of bacterial hosts prone to developing antibiotic resistance during infections [6,7,8,9].

The Food and Agriculture Organization of the United Nations and the WHO (FAO/WHO) define probiotics as “live microorganisms which when administered in adequate amounts confer a health benefit on the host,” and probiotic bacteria are now being developed for medical applications [10]. Traditionally, probiotic treatments have been associated with alleviating gastrointestinal disorders, such as those associated with *Helicobacter pylori* [11]. Recent proposals have included the use of probiotics in sanitizing healthcare environments [12] and employing detergents in conjunction with selected probiotics that can displace surrounding pathogens through competitive exclusion [13]. One interesting approach to control infections among crowded community environments is the use of probiotic-based sanitation [14]. This method is reportedly effective in reducing levels of fungal, bacterial, and viral pathogens as an alternative to traditional chemical disinfectants and, in one study, was even used to help sanitize a subway microbiome [15]. Such breakthroughs are particularly compelling; as an example, a high-risk, antibiotic-resistant strain of *Pseudomonas aeruginosa* was cultured from an urban water drain in a populated subway underpass, demonstrating the multitude of pathogens that can be found in various environments that negatively impact human and animal health [16].

Research tools used by investigators to genetically modify microbial genes have traditionally been implemented using techniques such as transposon mutagenesis screens to identify the mechanisms of specific genes in certain phenomena, including the thermoregulation of motility in organisms, for example, typhoidal Salmonella [17]. The development of high-throughput sequencing techniques [18] combined with CRISPR (clustered regularly interspaced short palindromic repeats)–Cas systems [19] has revolutionized researchers’ ability to study microbial–host interactions [20]. One of the more intriguing developments is the technique of the newly developed MetaEdit (Metagenomic Editing) approach, which allows for targeted insertion of large DNA sequences into the genomes of bacteria within the mouse gastrointestinal tract [21]. The procedure involves the utilization of optimized CRISPR-associated transposases delivered by a conjugative vector system to genetically engineer a diverse ecology of commensal bacteria directly to single-nucleotide genomic resolution. The goal is to genetically modify individual bacteria within natural communities among gigabases of a metagenomic bacterial population. Moreover, techniques such as precision microbiome programming for therapeutic applications may eventually become a reality [22]. Certainly, the above breakthroughs represent potentially monumental advances in metagenomics analyses; for now, however, investigators will continue to utilize CRISPR systems to study individual pathogens [23].

Diagnostic development for clinically important microorganisms remains an essential aspect of microbial research for highly specific, rapid pathogen detection [24]. CRISPR systems have been adapted for microbial detection assays, which include the Combinatorial Arrayed Reactions for Multiplexed Evaluation of Nucleic acids (CARMEN), used for multiplexed pathogen detection [25]. This assay has recently been updated to detect 23 blood-borne pathogens critical to clinical diagnostics and public health surveillance [26]. Another technique, matrix-assisted laser desorption/ionization time-of-flight mass spectrometry (MALDI-TOF MS), has been developed for the detection of microorganisms, and databases have been established to characterize pathogens [27,28]. The technique has been utilized for the rapid detection of pathogen-associated illnesses such as *Staphylococcus aureus* bloodstream infections [29] and fungal biotyping [30]. Point-of-care diagnostics utilizing molecular techniques are another critical aspect of infectious disease detection and monitoring [31]. Such testing is particularly important in locations where rapid, low-cost alternative diagnostics are needed as it does not require sophisticated equipment and has been recommended by the WHO for initial diagnosis. Consequently, methods such as the loop-mediated isothermal amplification (LAMP) technique offer a promising tool for the diagnosis of pulmonary tuberculosis in resource-limited regions of the world [32]. In addition to nucleic acid techniques, time-honored serological diagnosis of parasitic diseases, such as toxoplasmosis, remains vital in many parts of the world [33].

Exploration of microbiomes in numerous environments is an ever-evolving area of research. On a related note, *Time* magazine recognized Dr. Jeffrey I. Gordon for his contributions to the development of a therapeutic food designed to treat childhood malnutrition as one of the best inventions of 2025 (https://time.com/collections/best-inventions-2025/7318496/mdcf-2/, accessed on 20 December 2025 and https://medicine.washu.edu/news/time-magazine-names-therapeutic-microbiome-directed-food-a-best-invention-of-2025/, accessed on 20 December 2025. https://www-nature-com.oregonstate.idm.oclc.org/subjects/microbiome, accessed on 20 December 2025). Severe global malnutrition affects 14 million children under 5 years of age, and food insecurity remains an ongoing challenge for which therapeutic interventions are needed, representing a significant societal burden in low- and middle-income countries [34]. Importantly, Dr. Gordon and collaborators created an intervention with a microbiota-directed complementary food that was more efficacious than a ready-to-use supplementary food. The results provide investigators and health personnel with a path that will provide a basis for further testing of the human microbiome to identify biomarkers and better define treatment responses for malnutrition [35,36]. Bacteria represent the primary focus of the majority of microbiome studies. However, fungi also play important roles in gastrointestinal microbiomes, yet these organisms are not as widely studied in microbial ecology research. The basis of new applications may depend on expanding our knowledge of the evolutionary origins of the gut microbiome, which will also depend on deciphering the importance of the mycobiome [37].

Environmental microbiology is a far-reaching subject that includes a diverse array of research, including aquatic microbial systems, biofilms, plant–fungal relationships, how microbes play roles in restoration ecology, and how microbes impact a variety of insect and animal microbiomes. Within this field, bioremediation utilizing consortia of microbes holds promise as a tractable, commercially viable strategy to treat wastewater and contaminated marine or freshwater environments [38]. Moreover, these approaches may also contribute to the production of useful byproducts such as fertilizers [39]. To improve upon these approaches, progress in the world of bioinformatics will continue to play a crucial role in bacterial genomics and metagenomics [40]. Consequently, new user-friendly metagenomics analysis techniques, such as MetaXplore, have been developed as an interactive interface written in the R language to analyze complex microbial communities through high-throughput sequencing of marker gene amplicon data. This process includes determining alpha and beta diversity, taxonomic composition, differential abundance analysis, and identification of the core microbiome [41].

Discovery and research in virology will continue to play important roles in microbiological research. The impact of viral disease-causing agents, such as severe acute respiratory syndrome coronavirus 2 (SARS-CoV-2) [42], mechanisms by which human immunodeficiency virus type 1 (HIV-1) impacts host cellular pathways [43], avian influenza in agricultural products [44], and the need for mpox diagnostics [45], combined with a “host” of other issues in virology, will continue to be a focus during the future [46]. One important aspect of viral pathogenesis research is related to the emergence of long COVID. The emergence of long-term illness after SARS-CoV-2 infection was first reported in 2020, following reports of patients with infection-associated chronic conditions, which included mainstream media coverage and official recognition of the condition among the human population [47]. The condition is defined as “an infection-associated chronic condition that occurs after SARS-CoV-2 infection and is present for at least 3 months as a continuous, relapsing and remitting, or progressive disease state that affects one or more organ systems” that manifests as interstitial lung disease with cardiac complications [48]. Research into the causes of long COVID includes investigating persistent immune activation and proinflammatory responses [49] and potential B-cell dynamics among individuals with long COVID [50], amongst other mechanisms of the disease to aid mitigation of the syndrome [51].

Of particular interest in the future will be to monitor how artificial intelligence (AI) and machine learning impact microbiology and microbiome research [52]. Within the diagnostic laboratory, advancements in image analysis, also known as computer vision, help interpret digital images through comparison with annotated reference standards and curation of data for quality control analysis, two areas that could expedite and improve accuracy in clinical microbiology practices [53]. Natural Language Processing (NLP) is being used to analyze microbial interactions in gastrointestinal communities to predict issues such as irritable bowel disease (IBD) and diet-related effects by identifying distinct taxa [54]. Due to the complexities of artificial intelligence, machine learning, and microbial ecology, it is beneficial to have accessible guides that provide foundational information on these subjects [55,56]. Microorganisms are the most abundant living entities on our planet; they play major roles in organismal health, the environment, and in food systems. Consequently, research involving understanding the mechanisms of molecular microbiology will continue to be of utmost importance far into the future.

## References

[1] Kaneko T., Nakatsuka K., Tsuneda S. (2025). Phage Cocktails: State-of-the-Art Technologies and Strategies for Effective Design. FEMS Microbiol. Rev..

[2] Seal B.S., Drider D., Oakley B.B., Brüssow H., Bikard D., Rich J.O., Miller S., Devillard E., Kwan J., Bertin G. (2018). Microbial-derived products as potential new antimicrobials. Vet. Res..

[3] Miller W.R., Arias C.A. (2024). ESKAPE pathogens: Antimicrobial resistance, epidemiology, clinical impact, and therapeutics. Nat. Rev. Microbiol..

[4] Bianco G., Boattini M., Cricca M., Diella L., Gatti M., Rossi L., Bartoletti M., Sambri V., Signoretto C., Fonnesu R. (2024). Updates on the Activity, Efficacy and Emerging Mechanisms of Resistance to Cefiderocol. Curr. Issues Mol. Biol..

[5] Marino A., Stracquadanio S., Cosentino F., Maraolo A.E., Colpani A., De Vito A., Geremia N., Nicolosi A., Oliva A., Cacopardo B. (2025). Phage to ESKAPE: Personalizing Therapy for MDR Infections-A Comprehensive Clinical Review. Pathogens.

[6] Zhu G.S., Yang X.X., Zhang Y., Zhang X.Y., Lin L.B., Wang F. (2026). Bacteriophage lysin P26ly combats multidrug-resistant Escherichia coli O157 and methicillin-resistant Staphylococcus aureus via cell wall degradation and membrane disruption. Int. J. Biol. Macromol..

[7] Ndiaye I., Debarbieux L., Sow O., Ba B.S., Diagne M.M., Cissé A., Fall C., Dièye B., Dieng A., Diop A. (2025). Characterization of broad host range bacteriophages vKpIN31 and vKpIN32 against hospital-acquired Klebsiella pneumoniae in Dakar, Senegal. Sci. Rep..

[8] Echterhof A., Dharmaraj T., Blankenberg P., Targ B., Nguyen T.D., Bollyky P.L., Smith N.M., Blankenberg F.G. (2025). Whole-body distribution of three Pseudomonas phages characterized by a translational physiologically based pharmacokinetic model. Antimicrob. Agents Chemother..

[9] Wang C., Zhao S., Jiang H., Shi H., Li J., Zhao C., Huang H. (2025). Biological Characteristics and Genomic Analysis of Acinetobacter nosocomialis Lytic Phage XC1. Curr. Issues Mol. Biol..

[10] Vinderola G., Druart C., Gosálbez L., Salminen S., Vinot N., Lebeer S. (2023). Postbiotics in the medical field under the perspective of the ISAPP definition: Scientific, regulatory, and marketing considerations. Front. Pharmacol..

[11] Santacroce L., Topi S., Bottalico L., Charitos I.A., Jirillo E. (2024). Current Knowledge about Gastric Microbiota with Special Emphasis on Helicobacter pylori-Related Gastric Conditions. Curr. Issues Mol. Biol..

[12] Denkel L.A., Voss A., Caselli E., Dancer S.J., Leistner R., Gastmeier P., Widmer A.F. (2024). Can probiotics trigger a paradigm shift for cleaning healthcare environments? A narrative review. Antimicrob. Resist. Infect. Control.

[13] D’Accolti M., Soffritti I., Bini F., Mazziga E., Caselli E. (2024). Tackling transmission of infectious diseases: A probiotic-based system as a remedy for the spread of pathogenic and resistant microbes. Microb. Biotechnol..

[14] Soffritti I., D’Accolti M., Bini F., Mazziga E., Volta A., Bisi M., Mazzacane S., Caselli E. (2025). Probiotic-Based Approaches for Sustainable Control of Infectious Risk in Mass Transport: Current Data and Future Perspectives. Microb. Biotechnol..

[15] D’Accolti M., Soffritti I., Bini F., Mazziga E., Cason C., Comar M., Volta A., Bisi M., Fumagalli D., Mazzacane S. (2023). Shaping the subway microbiome through probiotic-based sanitation during the COVID-19 emergency: A pre-post case-control study. Microbiome.

[16] Libisch B., Ozoaduche C.L., Keresztény T., Bus A., Van Limbergen T., Posta K., Olasz F. (2025). Detection of the ST111 Global High-Risk Pseudomonas aeruginosa Clone in a Subway Underpass. Curr. Issues Mol. Biol..

[17] Shem-Tov R., Gal-Mor O. (2025). HilE mediates motility thermoregulation in typhoidal Salmonella serovars at elevated physiological temperatures. PLoS Pathog..

[18] Akintunde O., Tucker T., Carabetta V.J. (2025). The Evolution of Next-Generation Sequencing Technologies. High Throughput Gene Screening; Methods in Molecular Biology.

[19] Benz F., Beamud B., Laurenceau R., Maire A., Duportet X., Decrulle A., Bikard D. (2025). CRISPR–Cas therapies targeting bacteria. Nat. Rev. Bioeng..

[20] Echavarria Galindo M., Lai Y. (2025). CRISPR-based genetic tools for the study of host-microbe interactions. Infect. Immun..

[21] Gelsinger D.R., Ronda C., Ma J., Kar O.B., Edwards M., Huang Y., Mavros C.F., Sun Y., Perdue T., Vo P.L. (2025). Metagenomic editing of commensal bacteria in vivo using CRISPR-associated transposases. Science.

[22] Whitaker W.R., Russ Z.N., Stanley Shepherd E., Popov L.M., Louie A., Lam K., Zong D.M., Gill C.C.C., Gehrig J.L., Rishi H.S. (2025). Controlled colonization of the human gut with a genetically engineered microbial therapeutic. Science.

[23] Wang S., Ding Y., Rong H., Wang Y. (2024). The Development of a CRISPR-FnCpf1 System for Large-Fragment Deletion and Multiplex Gene Editing in Acinetobacter baumannii. Curr. Issues Mol. Biol..

[24] Jhaveri T.A., Weiss Z.F., Winkler M.L., Pyden A.D., Basu S.S., Pecora N.D. (2024). A decade of clinical microbiology: Top 10 advances in 10 years: What every infection preventionist and antimicrobial steward should know. Antimicrob. Steward. Healthc. Epidemiol..

[25] Ackerman C.M., Myhrvold C., Thakku S.G., Freije C.A., Metsky H.C., Yang D.K., Ye S.H., Boehm C.K., Kosoko-Thoroddsen T.F., Kehe J. (2020). Massively multiplexed nucleic acid detection with Cas13. Nature.

[26] Kamariza M., McMahon K., Kim L., Welch N.L., Stenson L., Allan-Blitz L.T., Sanders G., Eromon P., Muhammad A., Sijuwola A.E. (2025). Multiplexed detection of febrile infections using CARMEN. Nat. Commun..

[27] Lasch P., Beyer W., Bosch A., Borriss R., Drevinek M., Dupke S., Ehling-Schulz M., Gao X., Grunow R., Jacob D. (2025). A MALDI-ToF mass spectrometry database for identification and classification of highly pathogenic bacteria. Sci. Data.

[28] Xiong Q., Guan H. (2025). Application of matrix-assisted laser desorption/ionization time-of-flight mass spectrometry in clinical testing and diagnosis. Front. Cell. Infect. Microbiol..

[29] Boattini M., Guarrasi L., Comini S., Ricciardelli G., Casale R., Cavallo R., Costa C., Bianco G. (2025). Diagnostic methods and protocols for rapid determination of methicillin resistance in Staphylococcus aureus bloodstream infections: A comparative analysis. Eur. J. Clin. Microbiol. Infect. Dis..

[30] Hernández-Torres K., Torres-Mendoza D., Navarro-Velasco G., Cubilla-Rios L. (2025). Toward an Efficient Differentiation of Two Diaporthe Strains Through Mass Spectrometry for Fungal Biotyping. Curr. Issues Mol. Biol..

[31] Hassman A., Rouchka C., Sunino D., Espinal F.V., Youssef M., Casey R.R. (2024). Molecular Point-of-Care Assay Development: Design and Considerations. Curr. Protoc..

[32] Rosas-Diaz M., Palacios-Reyes C., Godinez-Aguilar R., Escalante-Bautista D., Alfaro Hernández L., Juarez-Islas A.P., Segundo-Ibañez P., Salas-Cuevas G., Olvera-Serrano Á., Hernandez-Martinez J.C. (2025). New Tool Against Tuberculosis: The Potential of the LAMP Lateral Flow Assay in Resource-Limited Settings. Curr. Issues Mol. Biol..

[33] Sołowińska K., Holec-Gąsior L. (2025). Development and Evaluation of Six Novel Recombinant GRA Proteins in Serodiagnosis of Human Toxoplasmosis. Curr. Issues Mol. Biol..

[34] UNICEF, United Nations Children’s Fund (UNICEF), World Health Organization (WHO), International Bank for Reconstruction and Development/The World Bank (2023). Levels and Trends in Child Malnutrition: UNICEF/WHO/World Bank Group Joint Child Malnutrition Estimates: Key Findings of the 2023 Edition.

[35] Hartman S.J., Hibberd M.C., Mostafa I., Naila N.N., Islam M.M., Zaman M.U., Huq S., Mahfuz M., Islam M.T., Mukherji K. (2024). A microbiome-directed therapeutic food for children recovering from severe acute malnutrition. Sci. Transl. Med..

[36] Zhou C., Hibberd M.C., Lee E.M., Pilgaard B., Vuillemin M., Kiehn E., Henrissat S., Crane M.A., Cheng J., Pfaff L. (2025). Glycoside hydrolase-mediated glucomannan catabolism in *Segatella copri*, a target of microbiota-directed foods for malnourished children. Proc. Natl. Acad. Sci. USA.

[37] Van Syoc E.P., Gomez A., Davenport E.R., Bordenstein S.R. (2025). Gut fungal profiles reveal phylosymbiosis and codiversification across humans and nonhuman primates. PLoS Biol..

[38] Mishra T., Tiwari P.B., Kanchan S., Kesheri M. (2025). Advances in Microbial Bioremediation for Effective Wastewater Treatment. Water.

[39] Butcher J., Villette C., Zumsteg J., Maurer L., Barchietto T., Rigo R., Floch K., Cseh A., Buchet S., Stintzi A. (2025). Microbial bioremediation of persistent organic pollutants in plant tissues provides crop growth-promoting liquid fertilizer. Nat. Commun..

[40] Baev V. (2025). Bioinformatics Research in Bacterial Genomics and Metagenomics. Curr. Issues Mol. Biol..

[41] Bel Mokhtar N., Asimakis E., Galiatsatos I., Maurady A., Stathopoulou P., Tsiamis G. (2024). Development of MetaXplore: An Interactive Tool for Targeted Metagenomic Analysis. Curr. Issues Mol. Biol..

[42] Gao Y., Ni P., Hua Y., Chen S., Zhang R. (2025). Impact of SARS-CoV-2 Variant NSP6 on Pathogenicity: Genetic Analysis and Cell Biology. Curr. Issues Mol. Biol..

[43] Mouzakis A., Petrakis V., Tryfonopoulou E., Panopoulou M., Panagopoulos P., Chlichlia K. (2025). Mechanisms of Immune Evasion in HIV-1: The Role of Virus-Host Protein Interactions. Curr. Issues Mol. Biol..

[44] Spackman E., Jones D.R., McCoig A.M., Colonius T.J., Goraichuk I.V., Suarez D.L. (2024). Characterization of highly pathogenic avian influenza virus in retail dairy products in the US. J. Virol..

[45] Liu B.M., Yang Z. (2025). An urgent need for diagnostic tools to address global mpox public health emergencies. J. Clin. Microbiol..

[46] Holmes E.C., Krammer F., Goodrum F.D. (2024). Virology-The next fifty years. Cell.

[47] Al-Aly Z., Davis H., McCorkell L., Soares L., Wulf-Hanson S., Iwasaki A., Topol E.J. (2024). Long COVID science, research and policy. Nat. Med..

[48] Ely E.W., Brown L.M., Fineberg H.V., National Academies of Sciences, Engineering, and Medicine Committee on Examining the Working Definition for Long Covid (2024). Long Covid Defined. N. Engl. J. Med..

[49] Aid M., Boero-Teyssier V., McMahan K., Dong R., Doyle M., Belabbaci N., Borducchi E., Collier A.-R.Y., Mullington J., Barouch D.H. (2025). Long COVID involves activation of proinflammatory and immune exhaustion pathways. Nat. Immunol..

[50] Korobova Z.R., Arsentieva N.A., Liubimova N.E., Batsunov O.K., Butenko A.A., Kokoeva A.E., Kucherenko N.G., Kashchenko V.A., Boeva E.V., Norka A.O. (2025). B Cell Dynamics and Transitional B Cells in Long COVID. Curr. Issues Mol. Biol..

[51] Peluso M.J., Deeks S.G. (2024). Mechanisms of long COVID and the path toward therapeutics. Cell.

[52] Wang X.-W., Wang T., Liu Y.-Y. (2025). Artificial Intelligence for Microbiology and Microbiome Research. arXiv.

[53] Dien Bard J., Prinzi A.M., Larkin P.M., Peaper D.R., Rhoads D.D. (2025). Proceedings of the Clinical Microbiology Open 2024: Artificial intelligence applications in clinical microbiology. J. Clin. Microbiol..

[54] Pope Q., Varma R., Tataru C., David M.M., Fern X. (2025). Learning a deep language model for microbiomes: The power of large-scale unlabeled microbiome data. PLoS Comput. Biol..

[55] Walsh C., Stallard-Olivera E., Fierer N. (2024). Nine (not so simple) steps: A practical guide to using machine learning in microbial ecology. mBio.

[56] Cao H., de la Fuente-Nunez C. (2025). Microbial Primer: Artificial intelligence for microbiologists. Microbiology.

